# The role of antibiotic-loaded bone cement in complicated knee arthroplasty: relevance of gentamicin allergy and benefit from revision surgery — a case control follow-up study and algorithmic approach

**DOI:** 10.1186/s13018-020-01855-8

**Published:** 2020-08-12

**Authors:** Benjamin Thomas, Maria Benedikt, Ali Alamri, Florian Kapp, Rainer Bader, Burkhard Summer, Peter Thomas, Eva Oppel

**Affiliations:** 1 Department of Dermatology and Allergology, Ludwig-Maximilians-University Munich, Frauenlobstrasse 9-11, 80337 Munich, Germany; 2 Department of Hand, Plastic, and Reconstructive Surgery, Burn Center, BG Trauma Center, University of Heidelberg, Ludwig-Guttmann-Strasse 13, 67071 Ludwigshafen, Germany; 3 Department of Anesthesiology and Intensive Care, Technical University of Munich, Ismaninger Strasse 22, 81675 Munich, Germany; 4 Department of Orthopaedics, University of Rostock, Doberaner Strasse 142, 18057 Rostock, Germany

**Keywords:** Gentamicin, Bone cement, Allergy, Total knee arthroplasty, Implant failure, Revision

## Abstract

**Abstract:**

**Background:**

Antibiotic-loaded (particularly gentamicin) bone cement (BC) is widely used in total joint arthroplasty (TJA) to prevent periprosthetic infections (PPIs), but may itself cause implant failure. In light of a complete lack in literature, the objective was to assess the clinical relevance of gentamicin allergy for failure of cemented total knee arthroplasties in 25 out of 250 patients with positive patch test reactions to gentamicin and otherwise unexplained symptoms by evaluating benefits from revision with change to gentamicin-free cement.

**Methods:**

Fifteen of these 25 patients and their treating orthopaedic surgeons agreed to a re-assessment. They were surveyed regarding interim course of therapy and symptoms, including re-assessment of the Knee Injury and Osteoarthritis Outcome Score (KOOS), and underwent follow-up clinical and radiographic investigations. The initial use of gentamicin-loaded BC was reaffirmed by review of the primary implantation operative reports and respective implant passports. Primary and follow-up KOOS scores were analyzed regarding benefits from revision surgery by comparing nine patients with revision to six without revision.

**Results:**

Mean follow-up time was 38 months. The entirety of patients experienced an improvement of self-reported symptoms, with revision surgery (i.e., switching to gentamicin-free BC or uncemented total knee arthroplasty) yielding significantly greater improvement (*p* = 0.031): the nine revised patients reported a significant symptom relief (*p* = 0.028), contrary to the six unrevised patients (*p* = 0.14). Interestingly, the decision to proceed with revision surgery was significantly correlated with higher symptom severity (*p* = 0.05).

**Conclusion:**

In symptomatic total knee arthroplasty with gentamicin allergy, uncemented revision arthroplasty or change to gentamicin-free BC provides significant symptom relief.

## Background

Total joint replacement (TJR) is efficient and successful in the treatment of degenerative and inflammatory joint disorders: TJR can restore joint function, alleviate pain, and help regain quality of life. Therefore, total hip (THR) and knee replacement (TKR) have been acknowledged as the gold standard in advanced symptomatic osteoarthritis of the hip and knee joint. Today, approximately one million TJRs are implanted in the USA per year, with numbers projected to quadruple within the next two decades [1, 2]. However, there is a serious, yet frequent drawback to this success story: 9% of all TJR surgeries are complication-related revisions [3]. With an annual incidence of 1 to 3%, periprosthetic infection (PPI) brings about particularly poor clinical outcomes and technically difficult revision surgery [4, 5]. Moreover, the treatment costs of PPIs increase fourfold compared to primary implantations [6]. Alarmingly, the annual economic burden of infectious joint revisions is projected to exceed $1.5 billion by the end of 2019 [7]. Understandably, many attempts have been undertaken to reduce the incidence of PPIs, such as administering systemic antibiotics, or the use of antibiotic-loaded bone cement (ALBC).

Nowadays, poly-methylmethacrylate (PMMA)-based bone cement (BC) is the most widely used antibiotic vehicle in this context. This BC comprises a two-part system composed of a powdery and a liquid component. The former contains an acrylic MMA-polymer and an initiator (e.g., benzyl peroxide [BPO]) to start the redox polymerization, and the latter contains an acrylic MMA-monomer, an activator to maintain the polymerization reaction (e.g., *N*,*N*-dimethyl-p-toluidine [DMPT]), and a stabilizer to prevent premature curing (e.g., hydroquinone) [8, 9]. Based on the premise of enhanced prostheses fixation and prophylactic antibiotic delivery, standard use of ALBC has been common practice in the UK [10, 11], Scandinavia [12], and Germany [13]. On the contrary, despite representative polls reporting an increasing use of primary prophylactic ALBC [14], the majority of orthopaedic surgeons in the USA remain skeptical [15].

Regarding the assumption of increased arthroplasty fixation, the literature is inconclusive for cementing joint replacements. On the one hand, cemented THRs exhibit superior long-term as well as short-term survival [16]. On the other hand, however, whether cemented TKRs yield superior overall survivorship remains uncertain [16]. Likewise, the antimicrobial efficacy of ALBC has not been convincingly demonstrated. Despite strong evidence of high local antibiotic concentrations, both immediately after implantation and sustainably over several months [17–19], its clinical benefit remains controversial: while some meta-analyses report a significantly reduced PPI rate in primary and revision arthroplasty [20, 21], others found no effect [22, 23]. This discrepancy is further aggravated by several key disadvantages, such as the development of antibiotic-resistant bacterial strains [24, 25], thermal injury to surrounding tissues [26, 27], economical expenses for hospitals and tax payers [28], and the risk of allergenic potential to surgeons and patients [29–31].

We recently reported on 250 patients who had been referred to our outpatient allergy clinic with a history of complicated cemented arthroplasty and suspected implant allergy [32]. After mechanical problem elicitors, such as loosening or malalignment, had been ruled out by the transferring orthopaedic surgeon by means of clinical and radiographic investigations, extended patch testing with implant metals and the additional BC series was carried out at our clinic. We found positive reactions in 138 patients (55%), in particular, 41/138 reactions (30%) to implant metals and 49/138 (36%) to BC components. Interestingly, gentamicin sulfate triggered positive readouts in 25/250 patients (10%), with 17/25 reactions (68%) only appearing as late as day 6. However, the clinical relevance of positive patch tests to BC components has merely been suggested in a handful of case reports [33–36], with literature on revisions in the context of gentamicin allergy lacking completely. Thus, we followed up with our patients in an effort to investigate the rate and outcomes of re-operations in this cohort. To this end, our present study is the first to objectify symptom improvement after TJR and ALBC exchange in the context of gentamicin allergy. Our data emphasize both the clinical importance of an adequate work-up in cases of suspected allergic implant failure and the benefit of revisional surgery in cases of confirmed gentamicin allergy.

## Methods

### Patient cohort

**Fig. 1:**
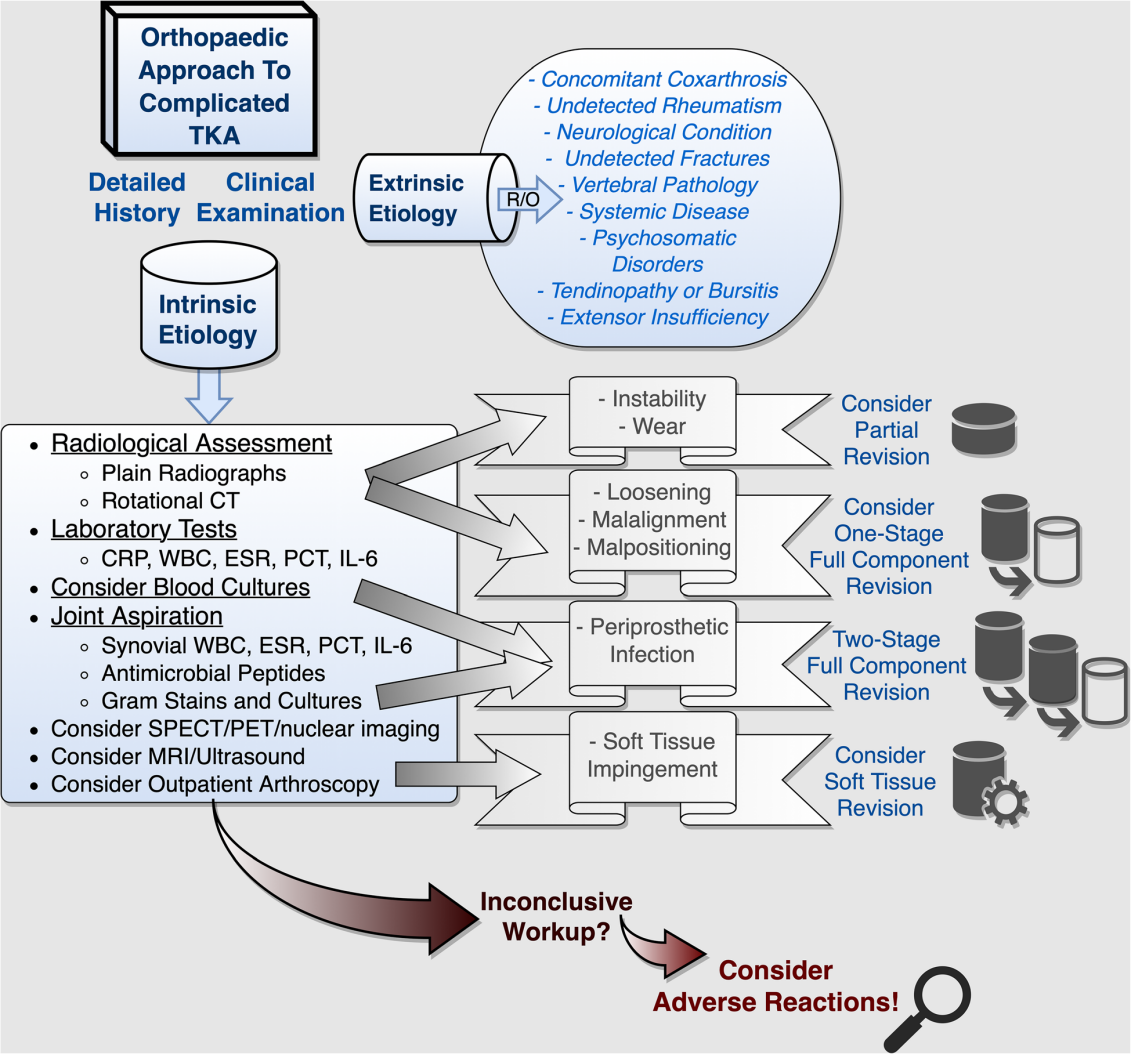
Traditional orthopaedic work-up of common problem elicitors. The initial work-up for complicated TKAs is carried out by the treating orthopaedic surgeon and commences with detailed history taking and a through clinical examination. Once systemic and extra-articular etiologies are ruled out, specific TKA-related problems are addressed. In cases of inconclusive work-up, rare conditions, such as allergic or adverse reactions, should be considered as diagnoses of exclusion (R/O, rule out; CT, computed tomography; CRP, C-reactive protein; WBC, white blood cell count; ESR, erythrocyte sedimentation rate; PCT, procalcitonin; IL-6 , interleukin-6, SPECT, single photon emission computed tomography; PET, positron emission tomography; MRI, magnetic resonance imaging)

**Fig. 2:**
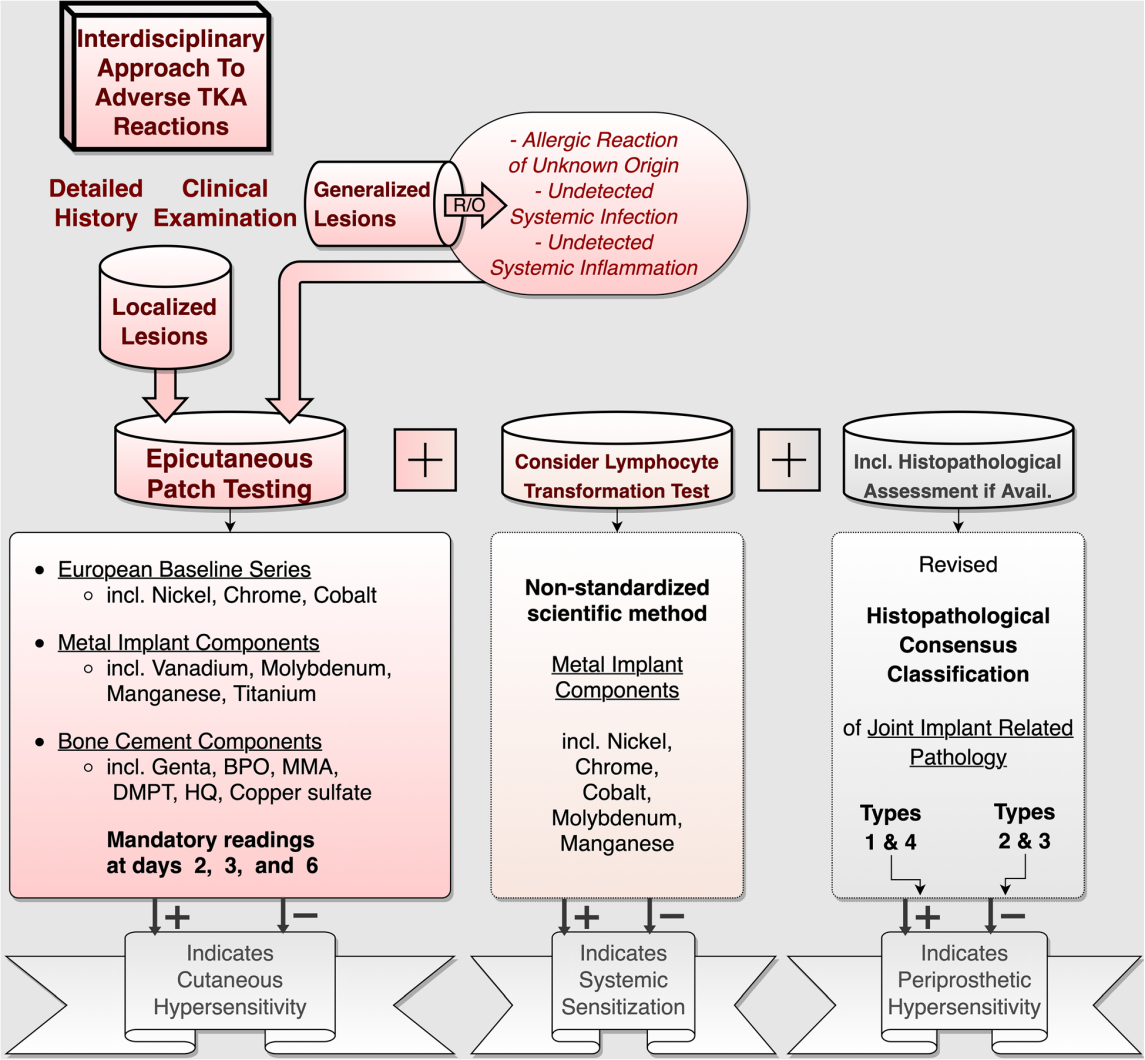
Algorithmic interdisciplinary approach to complicated TKAs with suspected allergic or adverse reactions. Following an inconclusive work-up for commonplace problem elicitors, symptomatic TKA-bearers should be sent for patch testing, particularly in cases of atopic diathesis or positive family history of allergy. After ruling out other systemic disorders, epicutaneous testing for relevant metal implant or bone cement hypersensitivities is carried out. This is typically accompanied by lymphocyte transformation testing for systemic sensitization and, in cases of earlier revisions or arthroscopic debridements, histopathological assessment (R/O, rule out; BPO, benzyl peroxide; MMA, methylmethacrylate; DMPT, *N,N*-dimethyl-*p*-toluidine; HQ, hydroquinone)

The present retrospective case series was approved by the local ethics committee (Reference number 159-14). By way of background, for over 20 years, we have been offering outpatient consultations for patients suspected of suffering from adverse implant reactions. Presenting with otherwise unexplained complicated and painful TJRs, dental and osteosynthetic materials, or other implants, such as cardiac pacers, these patients are referred to us by colleagues from a wide variety of fields. According to general consensus, we request that commonplace symptom elicitors be eliminated antecedently. In line with this, extrinsic factors are ruled out, and diagnostic approaches to potential articular etiologies are exhausted prior to consulting with us (Fig. 1). Once symptomatic patients have completed this initial orthopaedic routine work-up with inconclusive results, other systemic symptom elicitors are ruled out before specific allergic testing is commenced (Fig. 2). In order to establish the diagnosis of exclusion of an adverse implant reaction, a three-stage work-up algorithm is then followed: (1) Patch testing is initiated with three sets of substances (common allergens, metallic allergens, and bone cement components, see Tables 1 and 2). This is accompanied by lymphocyte transformation testing in order to check for acquired systematic sensitization as well as histopathological assessment of potential locoregional periprosthetic hypersensitivity.

**Table 1:** As part of the European baseline series (EBS) of haptens (contact allergens), 29 substances, prepared at different concentrations (conc.) in either a petrolatum (pet.) or aqueous (aq.) base (veh. = vehicle), are applied to the patient’s upper back in the form of a standardized commercially available patch test set

European baseline series	Allergen	Conc.	Veh.	Readings
	Potassium dichromate	0.5%	pet.	D2, D3, D6
	Thiuram mix	1.0%	pet.	D2, D3, D6
	Cobalt(II) chloride	1.0%	pet.	D2, D3, D6
	*Myroxylon pereirae*	25%	pet.	D2, D3, D6
	Colophonium	20%	pet.	D2, D3, D6
	*N*-isopropyl-*N*^′^ -phenyl-*p*-phenylenediamine	0.1%	pet.	D2, D3, D6
	Lanolin alcohol	30%	pet.	D2, D3, D6
	Mercapto mix	1.0%	pet.	D2, D3, D6
	Epoxy resin	1.0%	pet.	D2, D3, D6
	Nickel(II) sulfate	5.0%	pet.	D2, D3, D6
	*p*-tert-Butylphenol formaldehyde resin	1.0%	pet.	D2, D3, D6
	Formaldehyde	1.0%	aq.	D2, D3, D6
	Fragrance mix I	8.0%	pet.	D2, D3, D6
	Turpentine	10%	pet.	D2, D3, D6
	Methylchloroisothiazolinone/methylisothiazolinone	100 ppm	aq.	D2, D3, D6
	Paraben mix	16%	pet.	D2, D3, D6
	Cetearyl alcohol	20%	pet.	D2, D3, D6
	Zinc diethyldithiocarbamate	1.0%	pet.	D2, D3, D6
	Methyldibromo glutaronitrile	0.2%	pet.	D2, D3, D6
	Propolis	10%	pet.	D2, D3, D6
	Bufexamac	5.0%	pet.	D2, D3, D6
	Compositae mix II	5.0%	pet.	D2, D3, D6
	Mercaptobenzothiazole	2.0%	pet.	D2, D3, D6
	Hydroxyisohexyl 3-cyclohexene carboxaldehyde	5.0%	pet.	D2, D3, D6
	2-Bromo-2-nitropropane-1,3-diol	0.5%	pet.	D2, D3, D6
	Fragrance mix II	14%	pet.	D2, D3, D6
	Ylang Ylang (I + II) oil	10%	pet.	D2, D3, D6
	Sandalwood oil	10%	pet.	D2, D3, D6
	Jasmine absolute	5.0%	pet.	D2, D3, D6

**Table 2:** As part of the extended metal and bone cement series, 11 additional substances, each commercially available and prepared at different concentrations (conc.) in either a petrolatum (pet.) or aqueous (aq.) base (veh. = vehicle), are applied to the patient’s upper back in an individualized test set

Metal and bone cement series	Allergen	Conc.	Veh.	Readings
	Titanium(IV)-oxide	0.1%	pet.	D2, D3, D6
	Manganese(II)-chloride	0.5%	pet.	D2, D3, D6
	Molybdenum(V)-chloride	2.0%	pet.	D2, D3, D6
	Vanadium-pentoxide	10%	pet.	D2, D3, D6
	2-Hydroxyethylmethacrylate	1.0%	pet.	D2, D3, D6
	Copper(II) sulfate	1.0%	aq.	D2, D3, D6
	Benzoyl peroxide	1.0%	pet.	D2, D3, D6
	Gentamicin sulfate	20%	pet.	D2, D3, D6
	Hydroquinone	1.0%	pet.	D2, D3, D6
	*N,N-*Dimethyl*-p-*toluidine	2.0%	pet.	D2, D3, D6
	Methylmethacrylate	2.0%	pet.	D2, D3, D6

Amongst the previously tested 250 patients, all TKR bearers with positive patch test reactions to gentamicin were invited to a follow-up appointment at our clinic, as well as clinical and radiographic investigations carried out by the treating orthopaedic surgeons. Initial questionnaire-aided history, self-reported and questionnaire-aided anamnesis (including the German version of the Knee Injury and Osteoarthritis Outcome Score [KOOS]) [37, 38], and results from examinations for atopic diathesis were readily available, in addition to the primary clinical and radiographic orthopaedic examinations. An extended European baseline patch test series (Almirall Hermal, Reinbek, Germany), extended metal series (Chemotechnique Diagnostics, Vellinge, Sweden), and BC series had been tested as previously described [32], and readouts had likewise been documented in our database. Primary TKR fixation with gentamicin-loaded BC was reaffirmed by review of the respective operative reports and implant passports.

**Fig. 3:**
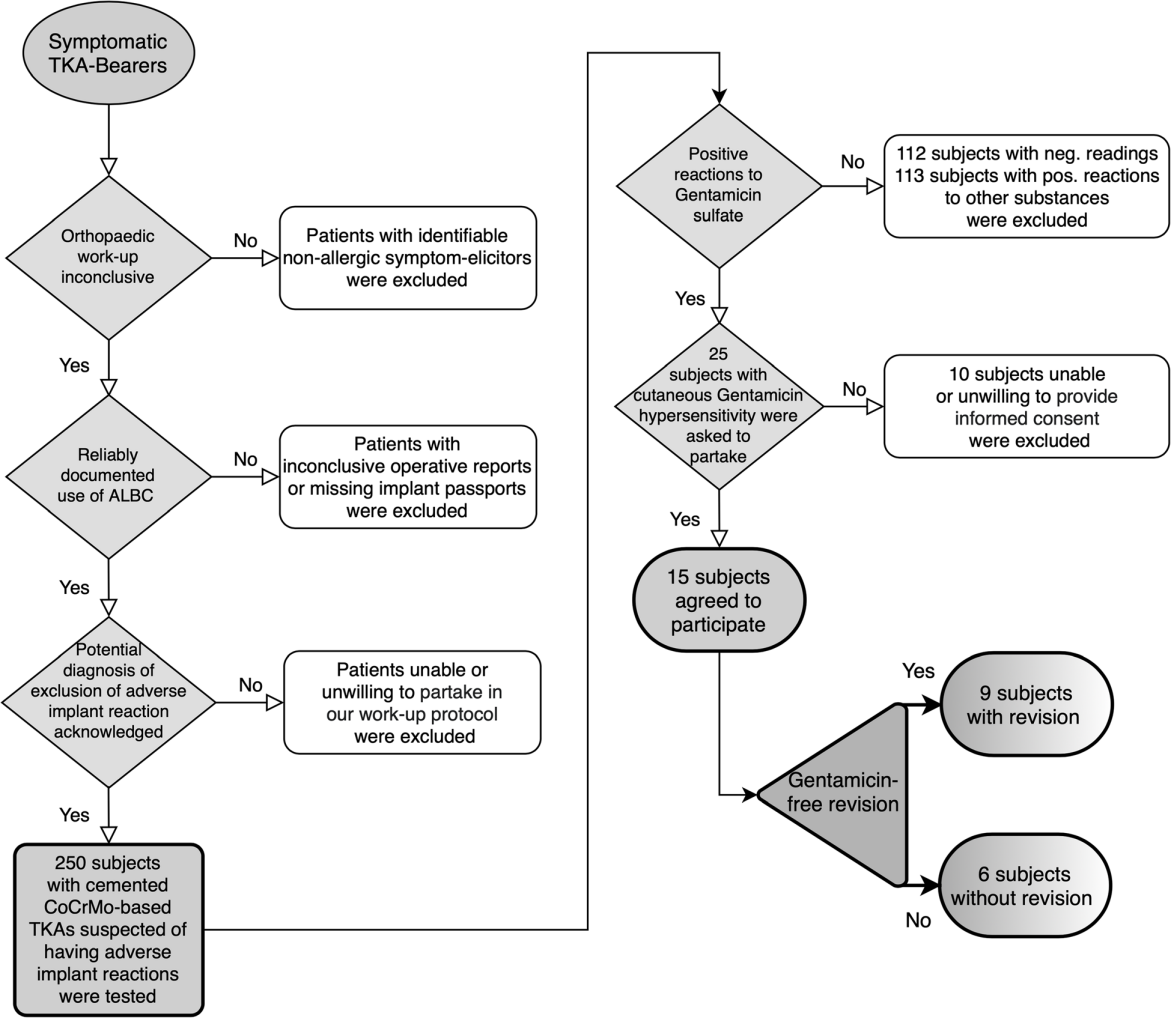
Flowchart of patient inclusion and exclusion criteria

Figure 3 illustrates the underlying patient inclusion/exclusion criteria for the present follow-up study.

### Follow-up procedure

After obtaining written informed consent, patients were reexamined by the referring orthopaedic surgeon and then re-assessed at our clinic by (i) means of a set of follow-up questions including potential revision surgeries with exchange of implant or BC components, (ii) recording any history of past and present implant-related complaints and the course of symptoms, and (iii) re-assessment of the KOOS Index. Based on the reported incidence of revision, patients were grouped into an “untreated’”(i.e., no revision with exchange of implant or BC components) and a “treated” cohort (i.e., revision with change to an uncemented TKA or re-implantation with gentamicin-free cement).

### Statistics

Statistical analysis was performed with IBM SPSS Version 20 (IBM Corporation, Armonk, NY, USA) and Graphpad Prism Version 7 (Graphpad Software, Inc., La Jolla, CA, USA). Primary and follow-up KOOS scores were compared using the paired *t* test to assess the differences in means. To assess the relationship between baseline KOOS scoring and the grouping variable (“treated” versus “untreated”), a one-tailed Pearson correlation coefficient was computed using a linear bivariate regression model. A two-way repeated-measures analysis of variance (ANOVA) was then run to determine whether the independent grouping variable (“treated” versus “untreated”) had an effect on KOOS score differences. An error probability of *p* ≤ 0.05 was considered statistically significant.

## Results

### Patient characteristics

**Fig. 4:**
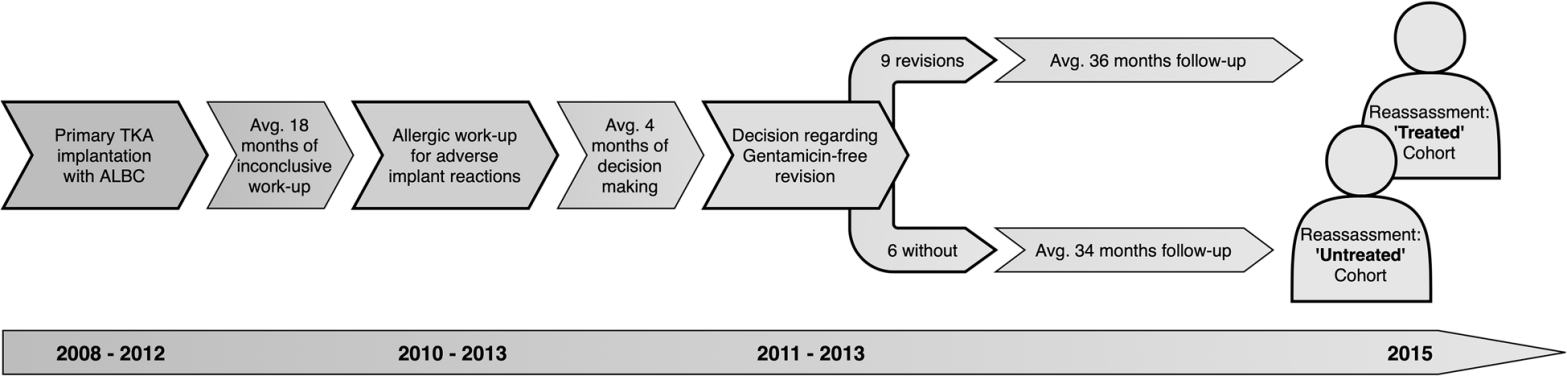
Timeline of patient recruitment and follow-up

Twenty-five symptomatic TKR bearers with proven gentamicin hypersensitivity were identified and invited to a follow-up interview, as well as clinical and radiographic investigations, with 15 of 25 allergic patients (60%) agreeing to partake in our study. The entire follow-up cohort of these 15 gentamicin allergic patients had initially been treated with cemented TKRs based on cobalt, chromium, and molybdenum (CoCrMo) alloy, which typically include ∼ 65% cobalt, ∼ 28% chromium, ∼ 6% molybdenum, and up to 1% nickel. The median age was 66 years (49–74 years), and 8 (53%) patients were female. All 15 patients had initially been referred to our clinic because of persistent and otherwise unexplained symptoms related to their TKR, after commonplace failure modes, such as bacterial infection, aseptic loosening, or malalignment had been excluded by the transferring orthopaedic surgeon prior to consultation with us. The mean implant to allergy work-up interval amounted to 18 months (4–53 months). At first visit, 12 patients complained of joint pain and limited mobility, respectively, 11 reported a history of recurrent swellings, and 7 noted intermittent joint effusions. Eczema (*n* = 3) and erythema (*n* = 1) were seldom stated (multiple complaints were reported per individual patient). Four patients had noticed previous adverse reactions (e.g., pruritus, erythema, and eczema) upon contact with metallic objects, such as wristwatches or jewelry. Furthermore, 6 patients reported a family history of atopic diathesis. None recalled “intolerance” reactions to topical drugs (including potential gentamicin-containing ointments, such as ear or eye drops). All patients had been found to be patch test positive—i.e., allergic—to gentamicin, with one patient reacting to both gentamicin and BPO. Moreover, two patients had combined reactions to gentamicin sulfate and nickel (II) sulfate. As part of our follow-study, re-assessment including repeated KOOS scoring was carried out after a mean follow-up period of 38 months (23–56 months). Figure 4 illustrates the chronological sequence of the aforementioned time intervals. In addition, a detailed summary of patient characteristics is given in Table 3.

**Table 3:** Patient and implant characteristics including date of implantation and implant type, allergy history, symptoms, patch test results, timing of allergy work-up and revision surgery, and change in KOOS scores (CoCroMo incl. Ni+, cobalt-chromium-molybdenum alloy includes up to 1% nickel; AA, allergic asthma; AR, allergic rhinitis; FA, family history of atopy; AE, atopic eczema; ARM, self-reported intolerance reactions to metallic objects; JP, joint pain; Sw, swelling; Er, localized erythema; Ec, localized eczema; JE, joint effusion; LA, limited ambulation; Genta, gentamicin; Ni, nickel; BPO, benzoyl peroxide)

ID	Implantation	Manufacturer: model	Alloy	Allergy history	Symptoms	Patch test	JR→WU †	JR→RS §	WU→FU £	KOOS Diff ‡
1	Apr 04, 2008	Zimmer: Protasul	CoCroMo (incl. Ni+)	none	JP, Sw, JE, LA	Genta	28	33	56	+ 62.1
2	Apr 09, 2009	DePuy: LCS	CoCroMo (incl. Ni+)	FA	JP, LA	Genta	19	22	54	+ 18.9
3	Apr 22, 2010	Smith and Nephew: Genesis II	CoCroMo (incl. Ni+)	FA	JP, Sw, JE, LA	Genta	11	15	52	+ 07.6
4	Jul 02, 2010	DePuy: PFC	CoCroMo (incl. Ni+)	FA	JP, Sw, JE, LA	Genta	13	17	45	+ 68.9
5	Apr 12, 2011	DePuy: PFC	CoCroMo (incl. Ni+)	AR, ARM	JP, Sw, Er, Ec, LA	Genta	4	no rev	45	+ 11.3
6	Mar 28, 2010	Biomet Oxford: Vanguard	CoCroMo (incl. Ni+)	AR, AA, AE	Sw, JE	Genta	19	no rev	43	− 04.6
7	Jan 21, 2011	Zimmer: INNEX	CoCroMo (incl. Ni+)	none	JP, Sw, LA	Genta	14	18	37	+ 33.4
8	Feb 28, 2011	DePuy: PFC	CoCroMo (incl. Ni+)	none	JP, Sw	Genta	13	17	37	+ 19.2
9	Feb 18, 2011	DePuy: PFC	CoCroMo (incl. Ni+)	none	Sw, LA	Genta	16	no rev	35	+ 01.5
10	Aug 06, 2009	Endoplant: Solution EPP	CoCroMo (incl. Ni+)	AR, AA, AE, FA	JP, Sw, LA	Genta	35	38	33	− 14.4
11	Sep 16, 2011	DePuy: PFC	CoCroMo (incl. Ni+)	none	JP, Ec, JE, LA	Genta, BPO	13	no rev	31	+ 03.8
12	Sep 09, 2008	DePuy: LCS	CoCroMo (incl. Ni+)	AR, ARM, FA	JP, Sw, LA	Ni, Genta	53	no rev	26	+ 56.0
13	Aug 20, 2012	Stryker: Triathlon	CoCroMo (incl. Ni+)	ARM	JP, Ec, JE, LA	Genta	6	no rev	26	+ 28.8
14	Aug 26, 2011	Zimmer: INNEX	CoCroMo (incl. Ni+)	FA	JP, Sw, JE, LA	Genta	19	27	25	+ 03.7
15	Jun 06, 2012	Zimmer: Nexgen	CoCroMo (incl. Ni+)	ARM	JP, Sw, JE, LA	Ni, Genta	12	15	23	+ 16.7

†Interval between primary implantation of joint replacement (JR) and allergy work-up (WU) in months

§Interval between primary implantation of joint replacement (JR) and revision surgery (RS) in months

£Interval between allergy work-up (WU) and follow-up (FU) in months

‡Individual KOOS index difference (absolute change)

### Decision to proceed with revision surgery

**Fig. 5:**
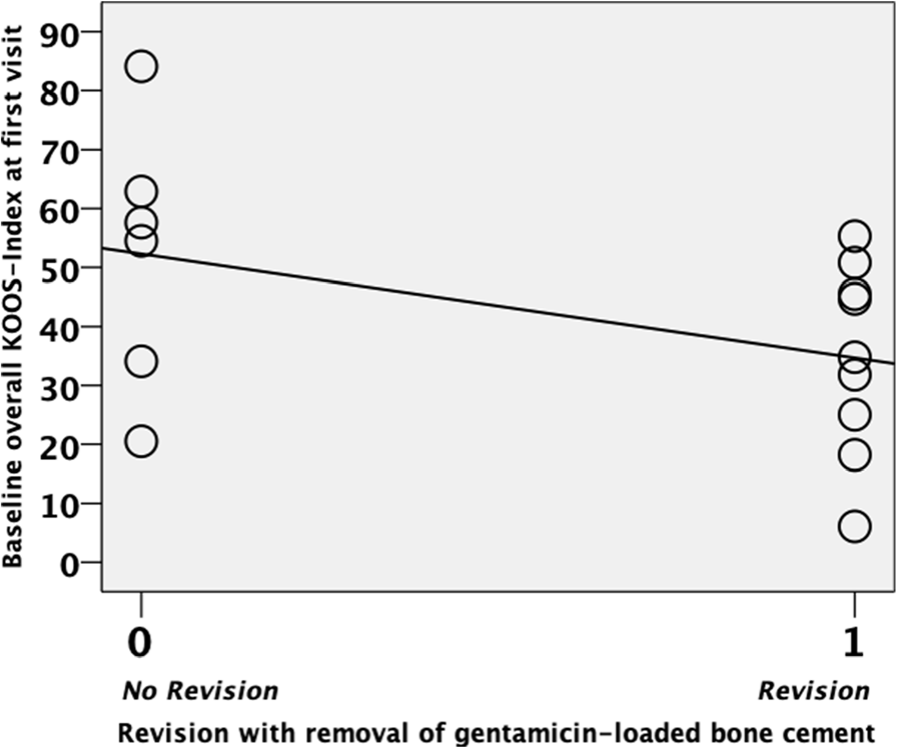
Correlation of KOOS scores upon first visit and the incidence of revision surgery. Baseline KOOS (Knee Injury and Osteoarthritis Outcome Score) indices of the 15 patients at first visit (i.e., time of allergy diagnostics). Scatterplot displaying the correlation of low KOOS indices (i.e., high symptom severity) and the decision to proceed with revision surgery in 9 patients (“revision”)

Overall, there was a significant difference between primary and follow-up KOOS scores for the entirety of all 15 patients (*t* = 3.258, *p* = 0.006, *n* = 15, 95% CI 7.124, 34.53), namely, less severe symptoms were reported at the time of our follow-up interview (*M* = 62.59, SD = 14.42) compared to the primary scoring (*M* = 41.73, SD = 20.19). A total of 9 patients had undergone revision surgeries with both the exchange of the TKR and removal of the gentamicin-loaded BC, corresponding to a “treatment” rate of 60%. Mean implant durability before revision was 22 months (15–38 months). Revision surgery was carried out after a mean interval of 4 months (2–7 months) following our diagnosis of gentamicin contact allergy. For all revisions, change to an uncemented TKA or re-implantation with gentamicin-free cement was confirmed by the operative report and implant passport. There was a significant negative correlation between baseline KOOS scores and the decision to proceed with surgery (*r* = − 0.442, *n* = 15, *p* = 0.050). By way of explanation, high degree of symptom severity (reflected by low KOOS scores) led to an increased probability of revision surgery. A scatterplot visualizes this relationship (Fig. 5). No unequivocal pathologic clinical (i.e., limited range of motion) or radiographic (i.e., malalignment or loosening) findings prompted any of the revision surgeries. In fact, our work-up results were indicated as the decisive criterion in favor of re-operation in all cases.

### Beneficial effect of gentamicin-free revision

**Fig. 6:**
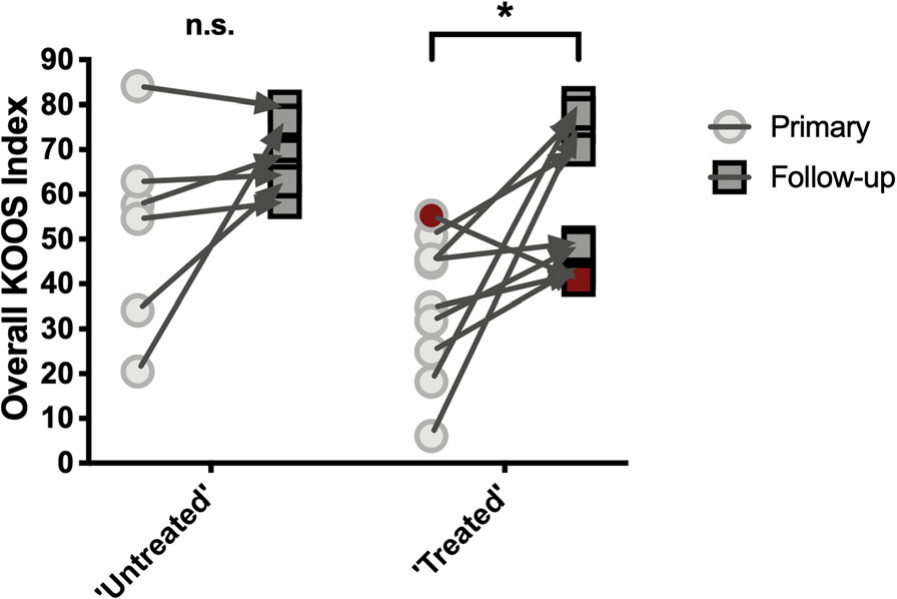
Revision surgery provides significant symptom relief in allergic patients. Split-plot repeated measures factorial ANOVA visualizing the improvement of KOOS (Knee Injury and Osteoarthritis Outcome Score) indices in the 6 patients without revision (“untreated”) and the 9 patients with revision (“treated”). Improvement was significant only in the “treated” group. Of note, patient 10 of the revision group displayed a score deterioration of 14.4 points (dark red). In this case, severe intra-articular arthrofibrosis was confirmed as the symptom elicitor by the treating orthopaedic surgeon. n.s., not statistically significant; *p < 0.05, primary, at first visit (i.e., time of allergy diagnostics)

Since we were not only interested in the motivation to proceed with revision, but also in the efficacy of these procedures, a repeated measures factorial ANOVA with “treatment status” as an independent binary factor and KOOS scoring as a repeated within-subjects factor was run. This analysis demonstrated a significant effect of the grouping variable (“treated” versus “untreated”) in the assumed direction (*F*(1, 13) = 5.877, *p* = 0.031), with significantly higher symptom relief after revision surgery (Figure 6). Further subgroup analysis using the paired t-test confirmed this significant improvement in the ‘treatment’ group (t = 2.627, p = 0.028, n = 9, 95% CI: 3.289, 44.73). In contrast, there was no significant change in KOOS scores for patients without revision (t = 1.748, p = 0.14, n = 6, 95% CI: -7.555, 39.65). Remarkably, one patient of the revision group, however, displayed a score deterioration of 14.4 points (highlighted in dark red in Fig. 6). Upon further investigation, the patient’s treating orthopaedic surgeon disclosed his diagnosis of severe intra-articular arthrofibrosis. This multi-faceted complication of TKR is characterized by excessive scar formation, low responsiveness to surgery, and poor functional and symptomatic outcomes [39].

## Discussion

Our follow-up investigation revealed that severely impaired TKA patients were more likely to not only acknowledge previously detected gentamicin allergy, but also to undergo revision surgery. Postoperative re-evaluation showed a significant improvement in KOOS scores in these patients—i.e., they benefited greatly from revision surgery. Since none of the traditional symptom elicitors had previously been detected (such as PPI or malalignment), allergy work-up was not only simply requested, but proven gentamicin allergy was in fact regarded as indication for revision surgery by the treating surgeons. This underlines the importance of a thorough allergy work-up in cases of otherwise unexplained TJR symptoms.

Indeed, up to 20% of TKA bearers remain dissatisfied and complain of persisting symptoms, such as joint stiffness, loss of range of motion, knee pain, and recurrent swelling or edema, following TKR [40, 41]. Despite a certain disagreement regarding the definition of arthroplasty failure in this context, almost a quarter of all TKA bearers undergoes surgical revision, mostly for aseptic loosening or PPI [42]. However, increasing numbers of revisions for unclear symptoms or following inconclusive work-up procedures are being recorded annually [43, 44]. It is in the context of these cases, that the present study is intending to raise awareness for rare problem elicitors, such as allergic or adverse reactions. As a matter of fact, only about 10% of orthopaedic surgeons report that they regularly screen their patients for relevant hypersensitivities [45].

Clinical observations of BC allergy as potential elicitor of arthroplasty failure have been reported as early as the 1970s [46, 47]: the allergens in question were acrylates, namely, methylmethacrylate (MMA) and additives, like BPO or DMPT. In 1996, Haddad et al. observed a high prevalence of positive patch test reactions to DMPT in a series of patients with early onset of aseptic implant loosening [48]. The importance of adequate preoperative history taking and patch testing—e.g., in patients with contact dermatitis to acrylic finger nails—has been discussed in a 2002 case report by Kaplan et al. [49] and a recent report by Alamri and colleagues [50]. Despite several reports focusing on BPO as an allergenic component in poorly tolerated cemented TJRs, its role in implant allergy remains controversial [51, 52]; BPO, despite its strong irritant potential, only acts as a weak allergen, resulting in false positive patch test reactions [53, 54]. Furthermore, its ongoing release from bone cement is questionable. This doubt is supported by the fact that BPO is metabolized rapidly as part of its function as redox initiator in the polymerization process [8, 9]. It is therefore doubtful whether BPO remnants are still present at the site of cementation after the curing process is complete. Nonetheless, Bircher et al. showed that removal of BC and change to uncemented endoprostheses resulted in considerable symptomatic improvements in 4 out of 5 BPO allergic patients with TJR [34]. Similarly, Vega et al. [36] and Kenan et al. [35] observed satisfactory long-term courses of uncemented revision arthroplasties in patients allergic to MMA, a BC component known to remain considerably longer at the site of implantation in cases of incomplete polymerization.

In our patients, however, the main reason for revision surgery was contact allergy to gentamicin sulfate, as confirmed by positive patch testing. Aminoglycoside antibiotics are well studied contact allergens [55], known to be responsible for contact dermatitis to topical drugs in particular, with gentamicin sulfate leading the way [56]. In line with this, a longitudinal study of 620 Finnish patients with suspected contact allergy revealed a 4.6% prevalence of positive patch test reactions to gentamicin [57]. However, our 15 patients did not report any intolerance or dermatitis to topical (potentially gentamicin containing) drugs. On the other hand, due to lifetime cumulative exposure, contact allergy to topical drugs becomes more prevalent with advancing age, which coincides with an increased demand for TJR [58]. However, reports of gentamicin allergy in cemented arthroplasty are scarce, apart from our preceding study [32]. Haeberle et al. presented a patient with a potentially gentamicin-related systemic contact dermatitis upon cemented TKR [59]. Christiansen et al. even reported a case of anaphylaxis with cardiac arrest after intravenous administration of gentamicin during a routine laparoscopic procedure, with thorough work-up revealing a previous cemented TKR as the suspected cause of sensitization [60]. Albeit devastating, the patient’s symptoms were of systemic nature and did not affect the TJR itself.

The abovementioned acute systemic reactions are starkly contrasted by the late onset and prolonged persistence of localized symptoms in our cohort of contact allergic patients. Allergic reactions in the sense of delayed type hypersensitivity may persist for a longer period of time even when continuous allergen exposure is low or has already stopped. This fact was well illustrated in an exemplary case by Wittman et al. [61], who described a gentamicin allergic patient with persistent and otherwise unexplained local symptoms following uncemented TKR. Meticulous history taking revealed a single intra-articular injection of gentamicin performed during an outpatient check-up as the elicitor of months-long local pain and swelling in this case. Of note, our results also demonstrate that in patients with less pronounced symptoms, complaints decreased as well, but they did so to a lesser degree. This might be explained by the gradually decreasing gentamicin release from BC over time.

Finally, our present study does have some weaknesses. First, and most importantly, our cohort only comprised a relatively small number of individuals, which complicates the generalizability of the conclusions drawn. Nevertheless, taking the large sample size of the primary study population into account, the numbers are sufficiently high to emphasize the importance of gentamicin contact allergy in complicated TJR. Secondly, our study is limited by its retrospective nature and involvement of several treating orthopaedic surgeons and their presumably slightly different treatment protocols, particularly with regard to their preference of considering positive patch test reactions as an indication for revision surgery. Third, we did not include serological investigations, histopathological sampling, or molecular methods to aid in the diagnosis of hypersensitivity. Thus, we were not able to compare the aforementioned diagnostic techniques in the context of suspected allergy to bone cement components. However, patch testing still remains the gold standard in detecting symptomatic contact allergy and was therefore deemed sufficient as the sole diagnostic criterion. Fourth, we did not analyze further patient characteristics, such as the pre-arthroplasty condition of their knees, potentially confounding comorbidities, or socioeconomic status, all of which might have contributed to their unsatisfactory primary TKA outcomes. Fifth, only 15 of all 25 patients diagnosed with gentamicin contact allergy were available for a follow-up visit, which might represent an inherent selection bias: namely, patients diagnosed with gentamicin contact allergy who did not profit from revision surgery might have refused to participate in our follow-up study, as opposed to those who did. Ultimately, we only used the KOOS to evaluate the course of symptoms and assess post-revision outcomes. Despite good evidence for reliability, validity, and responsiveness, the KOOS remains a solely self-reported score, omitting further objectifiable clinical and diagnostic criteria (such as range of motion or radiographic measurements) [62, 63]. One could therefore argue that an alternative scoring system, such as the New Knee Society Score, which contains a section of objective findings, would be better suited to determine the potential benefit of TKA revisions [64, 65]. However, despite these acknowledged weaknesses, our study provides encouraging evidence for the clinical relevance of an allergy work-up in cases of complicated TJR. These limitations do not weaken the strong correlation between gentamicin contact allergy and symptom improvement following revision surgery.

## Conclusion

With an ever-aging population, the demand of TJR is on the rise. At large, arthroplasty failure results in both a far-reaching physical and considerable financial burden on patients, physicians, and taxpayers. Consequently, trying to elucidate the mechanisms of premature implant failure remains a crucial multi-disciplinary challenge. In this context, particularly when symptoms persist despite an inconclusive work-up, rare conditions like implant allergy or adverse reactions should be considered as diagnoses of exclusion. In conclusion, our findings indicate that gentamicin allergy could represent a relevant problem elicitor in cemented arthroplasty. To our knowledge, this is the first study assessing the clinical relevance of gentamicin contact allergy in a single-center cohort of symptomatic patients with aseptic cemented TKR. To conclude, larger cohort studies, preferably multicenter in nature, as well as investigations of other joints, need to be performed to further corroborate our findings regarding the clinical relevance of gentamicin allergy in TKA.

## Data Availability

The datasets used and analyzed during the current study are available from the corresponding author on reasonable request.

## Abbreviations

ALBC: Antibiotic-loaded bone cement
ANOVA: Analysis of variance
BC: Bone cement
BPO: Benzyl peroxide
CoCrMo: Cobalt, chromium, and molybdenum
DMPT: *N*,*N*-Dimethyl-p-toluidine
e.g.: For example
i.e.: That is
KOOS: Knee Injury and Osteoarthritis Outcome Score
M: Mean/average
MMA: Methylmethacrylate
PMMA: Poly-methylmethacrylate
PPI: Periprosthetic infection
SD: Standard deviation
THR: Total hip replacement
TJA: Total joint arthroplasty
TJR: Total joint replacement
TKR: Total knee replacement
